# Buffalo Medical and Surgical Association

**Published:** 1884-05

**Authors:** Clayton M. Daniels

**Affiliations:** Secretary


### Society Reports.

### BUFFALO MEDICAL AND SURGICAL ASSOCIATION.

*Regular Annual Meeting, April 1st, 1884.*

President, Dr. John Cronyn, in the Chair.

Present — Drs. Fowler, Berkes, Moody, Dayton, Diehl, O'Brien, Phelps, J. C. Greene, Strong, Folwell, Hopkins, Johnson, Hauenstein, Hayd, Dorland, Bartlett, Mann, Tremaine, Howe, Rochester, Long, Hartwig, Heath, Frank, Weil, Fell, Runner, Brecht, F. H. Potter, Peterson, Davidson, Van Peyma, Putnam, Wm. Ring, W. W. Potter, Stockton, Hubbell, Dagenais, and, by invitation, Drs. Niemand, Hawkins, B. G. Long and Keene.

Applications for membership were received from Drs. B. G. Long and W. H. Thornton, who were duly elected.

Essay by Dr. M. D. Mann. Subject: "Axis Traction Forceps, with Exhibition of Instruments."

After the reading of the essay, the President suggested that as the subject was an important one, and it being the annual meeting, the time for discussion must necessarily be short. Considering the amount of business on hand, he thought it advisable to postpone the discussion until a future meeting.

Dr. Rochester thought that the discussion would, at best, be theoretical, as very few of the gentlemen present had had any experience with the instruments shown. Referring very briefly to his own experience, he felt certain he had seen the head perforated where it was unjustifiable, there being no pelvic deformity.

Dr. Hartwig felt that all could be done that was necessary with the ordinary instruments. He also remarked that some of Dr. Mann's drawings, as exhibited, were somewhat faulty.

Upon motion, it was decided to postpone the discussion to some future meeting.

*Prevailing Diseases*—Dr. Mann asked in regard to there being any puerperal fever in the city.

Dr. Brecht reported one case.

Dr. J. C. Greene said there were several in the lower part of the city.

*Miscellaneous Business*—Dr. Johnson made a few remarks regarding the bill to create a Board of Pharmacy. He said the bill had just passed the legislature and was now before the governor, and asked that this association endorse it by the following resolution:

"*Resolved,* That this association cordially endorse the Erie County Pharmacy Bill, and hope it may become a law."

Upon motion, the resolution was carried.

The Treasurer submitted his annual report, as follows:

| | |
|---|---|
| Cash on hand, April 3, 1883, - - - - - | $46.34 |
| Cash collected during year, - - - - | 92.05 |
| | $138.39 |
| Disbursements, - - - - - - - | 62.95 |
| Cash on hand April 1, 1884, - - - - | $75.44 |

F. E. L. Brecht, *Treasurer.*

Drs. Johnson, Greene and Heath were appointed an auditing committee to inspect the Treasurer's accounts.

The Secretary reported as follows:

*Mr. President and Members of the Association:*

In presenting my second annual report, I take pleasure in stating that the interest taken in our meetings during the past year has been a source of gratification and argues well for the future prospects of the association. Our membership has steadily increased and is now larger than ever before, being, at present, ninety-four, not including several who will be admitted as soon as qualified. The papers read have been of exceptional interest, and nearly all published in full.

I regret to say that the Treasurer reports some of our members, as yet, in arrears, and I take the liberty to suggest that it be made the duty of the Treasurer to prefer charges when dues are not paid as prescribed by the by-laws.

CLAYTON M. DANIELS, *Secretary.*

A vote of thanks was unanimously tendered the Secretary and Treasurer for services rendered the association.

*Election of Officers*—The following were duly elected for the ensuing year: President, F. W. Bartlett; Vice-President, C. G. Stockton; Secretary, Frederick Peterson; Treasurer, F. E. L. Brecht.

*Installation*—Upon retiring from the Chair, Dr. Cronyn congratulated his successor upon his preferment with a few pleasant and appropriate remarks.

Dr. Bartlett, in response, spoke of his grateful acknowledgement; also, referred to Dr. Cronyn's excellent qualities as a gentleman and presiding officer, and only hoped that at the end of the coming year it might be his good fortune to retire in as becoming a manner.

A unanimous vote of thanks was tendered Dr. Cronyn and ordered spread upon the minutes of the association.

Adjourned.

CLAYTON M. DANIELS, *Secretary.*

